# Portal vein thrombosis in laparoscopic vertical gastrectomy – laparoscopic sleeve gastrectomy: a case series

**DOI:** 10.1590/1677-5449.200013

**Published:** 2020-08-31

**Authors:** Jorge Oliveira da Rocha, Paula Dayana Matkovski, Fabrício Martins Zucco, Bernardo Dalago Ristow, Patrícia Moraes, Felipe José Koleski, Rinaldo Danesi Pinto, Flávio Silvério de Almeida Ponce

**Affiliations:** 1 Hospital Santa Catarina – HSC, Angioklinik, Cirurgia Vascular e Radiologia Intervencionista, Blumenau, SC, Brasil.; 2 Universidade Federal do Paraná – UFPR, Departamento de Ciências da Saúde, Curitiba, PR, Brasil.; 3 Hospital Santa Catarina – HSC, Vidar Clínica e Cirurgia, Blumenau, SC, Brasil.

**Keywords:** gastrectomy, venous thrombosis, morbid obesity

## Abstract

Grade III obesity is defined as excessive accumulation of fat in the body in a person with a BMI>40kg/m^2^ and is related to a series of comorbidities. It is therefore of fundamental importance that appropriate treatment is adopted to reduce its harmful effects on health. Laparoscopic vertical gastrectomy is well-established for treatment of grade III obesity. Although rare, portal vein thrombosis is one of the most serious of possible postoperative complications. In our study, eight cases are analyzed of laparoscopic vertical gastrectomy patients who developed portal vein thrombosis as a postoperative complication. In our series, we observed an increase in the incidence of portomesenteric venous thrombosis, especially among patients who did not follow the recommendations for oral hydration in the postoperative period. Most patients with this complication respond positively to anticoagulation, with complete or partial recanalization of the portal vein. Treatment with anticoagulants is effective and should be considered the first option. Vigorous hydration has also been shown to be an essential conduct in the postoperative period of these patients, and should always be encouraged.

## INTRODUCTION

Obesity is associated with systemic arterial hypertension, diabetes mellitus, degenerative joint diseases, gastroesophageal reflux, sleep apnea syndrome, chronic venous disorders, hypoventilation syndrome, abdominal wall hernias, and pseudotumor cerebri.^1^ Weight loss provoked by restrictive/malabsorptive surgery can lead to improvements in these comorbidities.^1^

Laparoscopic vertical gastrectomy (LVG) is well-established for treatment of grade III obesity.^2^ First described as a modification of the biliopancreatic diversion technique, LVG has achieved comparable results for long term weight loss and morbidity reduction to the Roux-en-Y technique.^3^^-^5

The majority of published series describe relatively low operative mortality associated with Roux-en-Y and vertical gastroplasty (around 1%).^6^ During the immediate postoperative period, morbidity is related to complications, such as infection of the surgical site, seroma, aponeurotic dehiscence, leaks or bleeding along the stapling and gastrojejunostomy lines, urinary infection, venous thromboembolism (VTE), and a range of pulmonary complications (atelectasis, respiratory infection, respiratory failure, and pulmonary embolism [PE]).^6^

The first report of portal vein thrombosis as a complication of LVG was published by Berthet et al.^7^ and was in a prothrombotic patient. Since Berthet’s report, other cases series were described,^8^ suggesting that this complication is not restricted to patients with thrombophilias.^9^

We present our experience of treating post-LVG portal vein thrombosis in a series of eight cases, covering the principal manifestations and the clinical results.

## DESCRIPTION OF THE CASES

In the period from January 2011 to December 2018, 1,347 LVG were performed and eight cases of portal vein thrombosis were diagnosed in these patients. All of the patients in our case series were operated at the same surgical center and by the same team of digestive apparatus surgeons, with proven experience in laparoscopic surgery.

**Figure 1:**
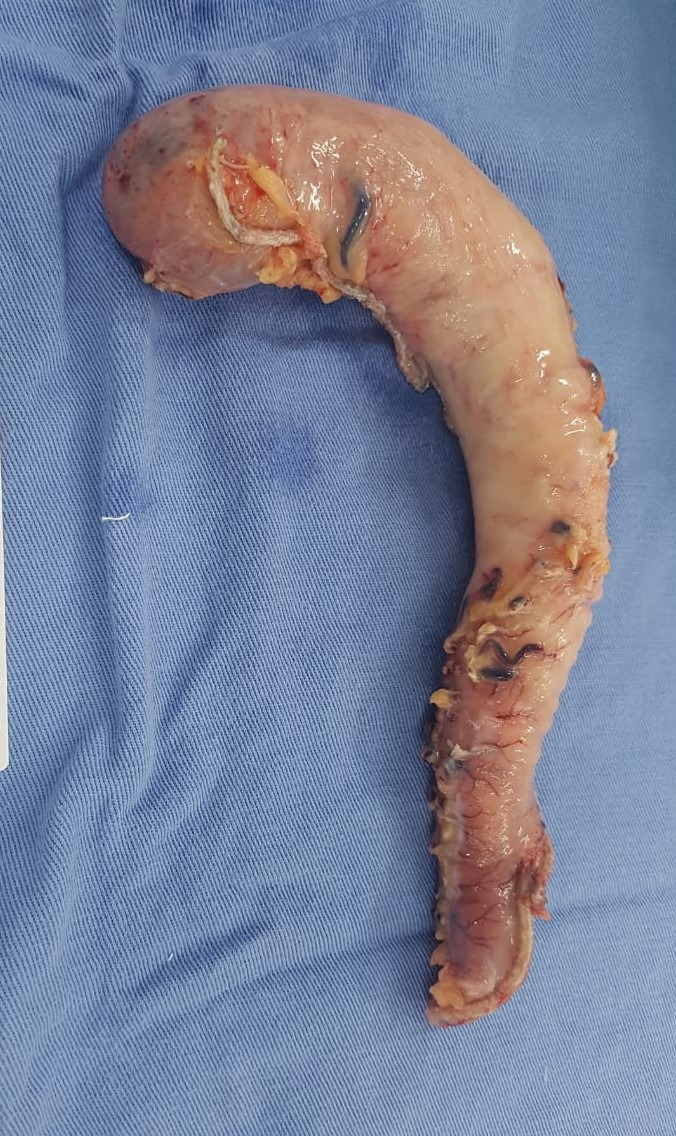
Photograph showing the surgical specimen on which LVG was performed. In this technique, vessels are sealed beyond a point 3 cm from the pylorus, working upwards flush to the gastric wall and within the gastroepiploic arch in the gastric body. The gastric fundus is released and the short gastric arteries are sealed. The stomach is thus vascularized by the left gastric artery only.

A five-port technique was used in all cases (Figure 1). During the surgery, patients were maintained in reverse Trendelenburg and pneumoperitoneum was established with CO_2_ at a pressure of 20 mmHg. After LVG, patients continued fasting for 8 hours and were then given water and tea in repeated small volumes.

In all cases, the Caprini score was employed as the TEV risk assessment model, and intermittent pneumatic compression (IPC) of the lower limbs was prescribed for 24 hours during the postoperative period, graduated compression elastic stockings were prescribed for 2 weeks, and prophylaxis with enoxaparin was administered at 80 mg/day in a single dose throughout the hospital stay and extended for a further 2 weeks after hospital discharge.

**Figure 2:**
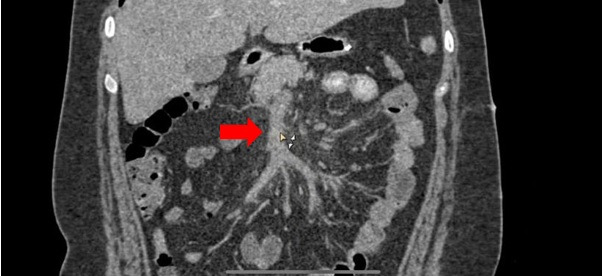
Photograph showing the portal phase of an abdominal angiotomography. The ectatic superior mesenteric vein can be seen with hypodense intraluminal content.

**Figure 3:**
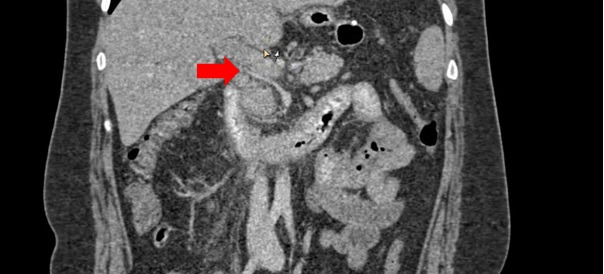
Photograph showing the portal phase of an abdominal angiotomography. The trunk of the ectatic portal vein can be observed with hypodense intraluminal content, characterizing portal vein thrombosis after laparoscopic vertical gastrectomy (LVG).

Patients with post-LVG portal vein thrombosis often have vague and nonspecific abdominal symptoms, such as nausea, abdominal distension and epigastric pain, which are common during the postoperative period after surgery on the digestive apparatus. Diagnoses of portal vein thrombosis were confirmed by abdominal angiotomography in the portal phase (Figures 2 and 3) in all patients who manifested any of these symptoms, even when nonspecific, looking for filling failures or increased caliber of the portal system vessels associated with an absence of contrast in the interior.

After diagnosis, all patients were transferred to the ICU and treated with unfractionated heparin (UFH), with an attack dose of 80 U/kg in bolus and a maintenance dosage of 18 U/kg/h, corrected according to the activated partial thromboplastin time (APTT), and vigorous hydration. Once the initial pain and abdominal distension had subsided, which took an average of 3 days, we moved to daily oral anticoagulation with warfarin sodium. Patients were discharged after achieving an INR (international normalized ratio) between 2.0 and 3.0.

There was one death among the cases described, which occurred at the start of our experience with treatment of this complication. We believe that the fatality occurred because of a premature laparotomy, with segmental enterectomy, and the lack of an adequate device for venous thrombectomy.

In the other seven cases treated with warfarin sodium, we observed total or partial recanalization of the portal vein, with development of a large network of collaterals and no need for any type of additional interventional procedure. Management consisted of conservative monitoring only, since none of the cases required elastic ligature of gastric or esophageal varices. The mean time on oral anticoagulation was 12 months, and INR was measured monthly, with warfarin sodium dosages adjusted as needed.

In one case, we observed inadvertent substitution of warfarin sodium for rivaroxaban by the patient herself, who had previously been treated with warfarin for 6 months. She suffered a lower digestive hemorrhage as a complication of having changed drugs. She was treated in hospital, with replacement of blood products and put back on warfarin sodium. The bleeding stopped and she did not suffer any further adverse events or need surgical reintervention.

## DISCUSSION

The most common complications of conventional surgery (open gastroplasty), are deep venous thrombosis (DVT), PE, pulmonary atelectasis, technical problems with anastomosis, hernias, and wound infections.^1^ In laparoscopic surgery, the most frequent complications are primarily those related to peritoneal distension, such as metabolic acidosis, cardiac arrhythmias, DVT, and PE.^1^

Portal thrombosis is an uncommon complication of surgeries involving the portal vein or mesenteric vein^2^ and is rare in laparoscopic surgery in general, although it can cause life-threatening conditions, such as ischemia or mesenteric infarction.^10^^-^14 The entire pathophysiology of portal vein thrombosis is not yet completely elucidated in these situations, but several factors are correlated. These include the reverse Trendelenburg, inflation under CO_2_ pressure, perioperative and postoperative dehydration, and also the prothrombotic status often seen in obese patients.^10^

In common with other reports of cases of portal vein thrombosis after LVG, we observed a delay before onset of symptoms, suggesting that there are other factors linked to this complication that are not limited to the intraoperative changes to visceral perfusion.^9^ Just as intra-abdominal sepsis has been blamed for spontaneous thrombosis of the portal vein,^15^ Csendes et al.^16^ consider that minor leaks along the stapling line are an initial presentation of portal vein thrombosis after laparoscopic gastrectomy, since both frequently occur during the same period.

In our case series, we observed an increase in the incidence of portomesenteric venous thrombosis, especially among those patients who did not follow the recommendations for oral route hydration during the postoperative period. After making this discovery, we adopted a policy of confirming water intake of at least 2 liters per day, and achieved total elimination of the incidence of this severe complication. We therefore believe that postoperative dehydration is an important etiologic factor in thrombosis of the portomesenteric system. The majority of patients respond positively to anticoagulation, with complete or partial recanalization of the portal vein, although for patients who exhibit progressive clinical deterioration, more invasive options such as percutaneous thrombectomy of the portal vein or thrombolysis techniques should be considered.^15^

We conclude that portomesenteric venous thrombosis is a rare, but severe, postoperative complication of treatment for grade III obesity in patients who undergo LVG. The symptoms of this complication are nonspecific, so a high degree of suspicion is needed to confirm diagnosis and initiate the appropriate treatment. Conservative treatment with anticoagulants has proven effective and should be considered the first option. Vigorous hydration is also an essential element of management of these patients during the postoperative period and must always be encouraged.
