# Supplementary material for: Portal vein thrombosis in laparoscopic vertical gastrectomy – laparoscopic sleeve gastrectomy: a case series
Source: J Vasc Bras. 2020 Aug 31;19:e20200013. doi: 10.1590/1677-5449.200013 (PMC8202208; doi:10.1590/1677-5449.200013)
Supplement: Translation

# Translation: Trombose da veia porta em gastrectomia vertical laparoscópica: série de casos

**Authors:** Jorge Oliveira da Rocha, Paula Dayana Matkovski, Fabrício Martins Zucco, Bernardo Dalago Ristow, Patrícia Moraes, Felipe José Koleski, Rinaldo Danesi Pinto, Flávio Silvério de Almeida Ponce

**Affiliations:** 1 Hospital Santa Catarina – HSC, Angioklinik, Cirurgia Vascular e Radiologia Intervencionista, Blumenau, SC, Brasil.; 2 Universidade Federal do Paraná – UFPR, Departamento de Ciências da Saúde, Curitiba, PR, Brasil.; 3 Hospital Santa Catarina – HSC, Vidar Clínica e Cirurgia, Blumenau, SC, Brasil.

**Keywords:** gastrectomia, trombose venosa, obesidade mórbida

## Abstract

A obesidade grau III é definida como acúmulo excessivo de gordura no corpo, caracterizada por IMC > 40kg/m^2^ e está relacionada a uma série de comorbidades, sendo, por isso, de fundamental importância a adoção de um tratamento adequado para reduzir os efeitos deletérios na saúde do indivíduo. A gastrectomia vertical laparoscópica está bem estabelecida para o tratamento. Entre as complicações pós-operatórias possíveis, a trombose da veia porta destaca-se, apesar de rara. Em nosso estudo, foram analisados oito casos de pacientes submetidos à gastrectomia vertical laparoscópica que apresentaram como complicação pós-operatória o desenvolvimento de trombose da veia porta. Observamos em nossa série o aumento na incidência de trombose venosa portomesentérica, especialmente nos pacientes que não seguiram as recomendações de hidratação via oral no pós-operatório. A maioria dos pacientes com essa complicação responde de maneira positiva à anticoagulação, com recanalização completa ou parcial da porta. O tratamento com anticoagulantes mostra-se eficaz, devendo ser considerado como primeira opção. A hidratação vigorosa também tem se mostrado conduta essencial no pós-operatório desses pacientes, devendo ser sempre estimulada.

## INTRODUÇÃO

A obesidade está associada a hipertensão arterial sistêmica, *diabetes mellitus*, doenças degenerativas das articulações, refluxo gastroesofágico, síndrome da apneia do sono, desordem venosa crônica, síndrome da hipoventilação, hérnias da parede abdominal e pseudotumor cerebral^1^. A perda de peso proporcionada pela cirurgia de restrição/má absorção pode resultar na melhora dessas comorbidades^1^.

A gastrectomia vertical laparoscópica (GVL) está bem estabelecida para o tratamento de obesidade grau III^2^. Primeiramente descrita como uma modificação da técnica de desvio biliopancreático, a GVL tem apresentado resultados de perda ponderal e morbidade em longo prazo comparáveis a técnica Y de Roux^3^^-^5.

A maioria das séries publicadas relata uma mortalidade operatória relativamente baixa para as gastroplastias em Y de Roux e verticais (em torno de 1%)^6^. No pós-operatório imediato, a morbidade se relaciona com complicações, como infecção do sítio cirúrgico, seroma, deiscência aponeurótica, vazamentos ou sangramento das linhas do grampeador e da gastrojejunostomia, infecção urinária, tromboembolismo venoso (TEV) e várias complicações pulmonares [atelectasia, infecção respiratória, insuficiência respiratória e embolia pulmonar (EP)]^6^.

O primeiro relato de trombose da veia porta como uma complicação da GVL foi feito por Berthet et al.^7^ em um paciente trombofílico. Desde o relato de Berthet, outras séries de casos foram reportadas^8^, sugerindo que essa complicação não é restrita apenas aos pacientes com trombofilias^9^.

Apresentamos nossa experiência no tratamento da trombose de veia porta em uma série de oito casos pós-GVL, bem como suas principais manifestações e resultados clínicos.

## DESCRIÇÃO DOS CASOS

No período compreendido entre janeiro de 2011 e dezembro de 2018, foram realizadas 1.347 GVL e, destas, oito casos de trombose de veia porta foram diagnosticados. Em nossa série de casos, todos os pacientes foram operados em um único centro cirúrgico e pela mesma equipe de cirurgiões do aparelho digestivo, com comprovada experiência em cirurgias laparoscópicas.

Utilizou-se, em todos os casos, a técnica de cinco Ports (Figura 1). Durante a cirurgia, os pacientes foram mantidos em proclive e com pneumoperitônio com CO_2_, utilizando uma pressão de 20 mmHg. Logo após a GVL, os pacientes permaneceram em jejum durante oito horas e, após esse período, receberam água e chá em quantidade fracionada.

**Figura 1:** Foto mostrando peça cirúrgica em que foi realizada a GVL. Nesta técnica, realiza-se selagem dos vasos a partir de 3 cm do piloro, subindo rente a parede gástrica e por dentro da arcada da gastroepiplóica no corpo gástrico. Libera-se o fundo gástrico, selando os gástricos curtos. O estômago fica vascularizado somente pela artéria gástrica esquerda.

Em todos os casos, aplicou-se o escore de Caprini como modelo da avaliação de risco de TEV, sendo prescritos no pós-operatório a compressão pneumática intermitente (CPI) dos membros inferiores durante 24 horas, o uso de meias elásticas de compressão graduada por duas semanas e a farmacoprofilaxia com enoxaparina 80 mg/dia em dose única durante todo o período da internação e estendida por mais 2 semanas após a alta hospitalar.

Frequentemente, os pacientes com trombose da veia porta pós-GVL apresentam sintomas abdominais vagos e inespecíficos, como náuseas, distensão abdominal e dor epigástrica, que são comuns no pós-operatório de cirurgias do aparelho digestivo. O diagnóstico da trombose da veia porta foi confirmado por angiotomografia de abdômen na fase portal (Figuras 2 e 3) em todos os pacientes que apresentavam algum desses sintomas, mesmo inespecíficos, observando-se falhas de enchimento ou aumento do calibre dos vasos do sistema portal associados à ausência do contraste no seu interior.

**Figura 2:** Foto da fase portal da angiotomografia de abdômen. É possível visibilizar a veia mesentérica superior ectasiada e com conteúdo hipodenso intraluminal.

**Figura 3:** Foto da fase portal da angiotomografia de abdômen. Observa-se o tronco da veia porta ectasiada e com conteúdo hipodenso intraluminal, caracterizando trombose de veia porta pós-gastrectomia vertical laparoscópica (GVL).

Após o diagnóstico, todos os pacientes foram encaminhados para a UTI e tratados com heparina não-fracionada (HNF), com dose de ataque de 80 U/kg em *bolus*, e dose de manutenção de 18 U/kg/h, corrigida de acordo com o tempo de tromboplastina parcial ativada (TTPA) e a hidratação vigorosa. Com a melhora do quadro álgico inicial e diminuição da distensão abdominal, que em média durou 3 dias, iniciamos a anticoagulação oral com varfarina sódica diariamente. Os pacientes receberam alta após atingir a INR (razão normativa internacional) entre 2,0 e 3,0.

Dentre todos os casos relatados, houve um óbito, que ocorreu no início de nossa experiência no tratamento dessa complicação. Acreditamos que a fatalidade ocorreu devido à laparotomia precoce, com enterectomia segmentar, e à indisponibilidade de dispositivo adequado de trombectomia venosa.

Nos outros sete casos tratados com varfarina sódica, observamos a recanalização total ou parcial da veia porta, com surgimento de grande rede de colaterais, sem necessidade de qualquer procedimento terapêutico intervencionista adicional, sendo feito apenas tratamento expectante, pois nenhum caso necessitou de ligadura elástica de varizes gástricas ou esofágicas. O tempo médio de anticoagulação oral foi de 12 meses, sendo realizadas mensurações mensais de INR, com ajustes das doses de varfarina sódica, quando necessário.

Observamos, em um único caso, a mudança inadvertida pelo próprio paciente da varfarina sódica para a rivaroxabana, tendo a paciente sido tratada anteriormente por 6 meses com varfarina. Essa paciente apresentou hemorragia digestiva baixa como complicação da alteração medicamentosa. Ela foi tratada em regime de internação, com reposição de hemocomponentes e retorno ao uso de varfarina sódica, com cessação do sangramento e ausência de novos eventos adversos, assim como da necessidade de nova intervenção cirúrgica.

## DISCUSSÃO

Na cirurgia convencional (gastroplastia aberta), as complicações mais comuns são a trombose venosa profunda (TVP), EP, atelectasia pulmonar, problemas técnicos na anastomose, hérnias e infecção na ferida^1^. Na cirurgia laparoscópica, as complicações mais frequentes são principalmente aquelas relacionadas com a distensão peritoneal, como acidose metabólica, arritmias cardíacas, TVP e EP^1^.

A trombose portal é uma complicação pouco frequente nas cirurgias envolvendo a veia porta ou mesentérica^2^ e rara nas cirurgias laparoscópicas em geral, podendo causar condições ameaçadoras à vida, como a isquemia ou o infarto mesentérico^10^^-^14. A fisiopatologia completa da trombose da veia porta nessas situações ainda não está completamente elucidada, embora vários fatores se correlacionem. Dentre eles, incluem-se o proclive, insuflação sob pressão do CO_2_, desidratação peri e pós-operatória, além de *status* pró-trombótico frequentemente encontrado em pacientes obesos^10^.

À semelhança dos relatos de caso de trombose da veia porta após GVL, observamos uma demora no início dos sintomas, sugerindo outros fatores ligados a essa complicação que não somente as alterações intraoperatórias da perfusão visceral^9^. Assim como a sepse intra-abdominal tem sido responsabilizada pela trombose espontânea da veia porta^15^, Csendes et al.^16^ consideram pequenos vazamentos na linha de grampeamento como a apresentação inicial da trombose da veia porta após gastrectomia laparoscópica, visto que ambos ocorrem frequentemente no mesmo período.

Observamos em nossa série de casos um aumento na incidência de trombose venosa portomesentérica, especialmente naqueles pacientes que não seguiram as recomendações de hidratação via oral no pós-operatório. Após essa constatação, adotamos a conduta de corroborar a ingestão hídrica de pelo menos 2 litros diários, obtendo a redução completa na incidência dessa grave complicação. Dessa maneira, acreditamos que um importante fator etiológico na trombose do sistema portomesentérico seja a desidratação pós-operatória. A maioria dos pacientes responde de maneira positiva à anticoagulação, com recanalização completa ou parcial da veia porta, enquanto, para os pacientes que apresentam piora clínica progressiva, opções mais invasivas como a trombectomia percutânea da veia porta ou técnicas de trombólise devem ser consideradas^15^.

Concluímos que a trombose venosa portomesentérica é rara, embora seja uma grave complicação pós-operatória no tratamento da obesidade grau III em pacientes submetidos à GVL. Os sintomas dessa complicação são inespecíficos, e um alto índice de suspeição é necessário para confirmar o diagnóstico e iniciar o tratamento adequado. O tratamento conservador com anticoagulantes tem se mostrado eficaz, devendo ser considerado como primeira opção. A hidratação vigorosa também é uma conduta essencial no pós-operatório desses pacientes, devendo ser sempre estimulada.
